# Emotional Intelligence as a Differentiated Personal Resource: Component-Level Associations with Burnout Among Higher-Education Teachers

**DOI:** 10.3390/bs16071154

**Published:** 2026-07-09

**Authors:** László Balázs

**Affiliations:** Department of Organizational Development and Communication Science, Institute of Social Sciences, University of Dunaújváros, 2400 Dunaújváros, Hungary; balazsl@uniduna.hu

**Keywords:** emotional intelligence, burnout, self-regulation, personal resources, job demands–resources model

## Abstract

Drawing on the personal-resource logic of the Job Demands–Resources (JD–R) model, this study examines emotional intelligence (EI) not as a single global trait but as a differentiated set of self-regulatory resources associated with distinct burnout dimensions. Using cross-sectional, self-report data from higher-education instructors (*N* = 292; complete-case *N* = 161–185 for the multivariable models), EI was assessed with the Bar-On Emotional Quotient Inventory and burnout with the BO-SE Burnout Screening Scales (mental, emotional, physical, and social exhaustion, plus an aggregated index); hierarchical regression, relative-importance analysis, and bootstrapped indirect-effect models were estimated. Overall EI was negatively associated with all burnout dimensions. At the component level, intrapersonal skills and stress management (stress tolerance and impulse control) were the two most consistent correlates, with complementary coverage—stress management most consistent for mental, emotional, and physical exhaustion, and intrapersonal skills strongest for mental and emotional exhaustion—while general mood was specific to physical exhaustion; associations survived false discovery rate correction. General mood and stress-management capacity statistically accounted for part of these associations in exploratory indirect-effect models. Because the design is cross-sectional and based on a single self-report method, findings are interpreted as associations and statistical indirect effects rather than causal mechanisms. The findings are consistent with a differentiated resource interpretation of EI in emotionally demanding work, but they do not establish causal mechanisms or temporal ordering.

## 1. Introduction

From an organizational functioning perspective, burnout is not merely an individual well-being concern but a work-related psychological state characterized by persistent exhaustion, mental distancing from work, and reduced professional efficacy, with substantial implications for performance, retention, and organizational sustainability (Maslach & Leiter, 2016; Schaufeli et al., 2020). Within contemporary work environments, one of the most influential explanatory frameworks in organizational behavior research is the Job Demands–Resources (JD–R) theory, which emphasizes the joint and dynamic role of job demands and resources in the development of burnout (Bakker & Demerouti, 2017; Bakker et al., 2023).

An important extension of the JD-R model and the JD-R framework lies in the extension of the resource construct beyond organizational aspects (autonomy, support) to personal resources, which are seen as core aspects of the resource construct (Hobfoll et al., 2018; Bakker & Demerouti, 2017; Van den Broeck et al., 2017). Personal resources comprise individual characteristics that may help employees appraise and manage the emotional and cognitive demands of the work environment and are often negatively associated with burnout indicators (Van den Broeck et al., 2017). In this context, emotional intelligence (EI) can be seen as an individual-level self-regulatory infrastructure, beyond the general concept of EI as a personal trait, to cope with enduring aspects of the work environment (Peña-Sarrionandia et al., 2015; Buruck et al., 2016). By a differentiated self-regulatory resource, we mean a set of functionally distinct but empirically correlated emotional-regulatory capacities (e.g., self-awareness, stress regulation, mood maintenance) that relate to different facets of strain, as opposed to a single global EI score.

Empirical research findings related to the relationship between EI and burnout show that EI is negatively correlated with burnout among different occupational groups (Gong et al., 2019; Almeneessier & Azer, 2023). For example, a study conducted in a South Asian context revealed that EI is negatively correlated with burnout symptoms (Han et al., 2022). Similarly, a study conducted among healthcare practitioners revealed that EI is a predictor of reduced burnout and increased well-being (Naggar et al., 2025). At the same time, other researchers revealed that contextual conditions, such as violence, might partially mediate the EI-burnout relationship, which suggests that environmental contingencies play a role in EI and burnout (Cao et al., 2022).

Higher-education instructors provide a particularly informative context, as academic teaching involves high and structurally embedded emotional demands and personal regulatory resources are especially salient in burnout-related strain (Balázs, 2020). Three gaps limit this literature, however. First, EI is most often modeled as a single global trait, so it remains unclear which specific regulatory capacities matter for burnout. Second, burnout is typically treated as a unitary outcome, obscuring whether different EI capacities map onto different forms of exhaustion. Third, the affective-regulatory processes assumed to link EI to burnout are rarely examined explicitly (Peña-Sarrionandia et al., 2015; Gong et al., 2019). Addressing these gaps calls for a component-level model of EI, a multidimensional model of burnout, and an examination of whether the observed data are consistent with differentiated indirect-effect patterns.

The present study addresses a theoretically relevant gap: how are specific components of emotional intelligence associated with distinct burnout symptoms in the workplace? Integrating emotion regulation research with the EI tradition suggests that EI may show both direct associations and indirect statistical relationships involving affective components (Peña-Sarrionandia et al., 2015). Meta-analytic evidence further supports the close association between EI and key emotion regulation processes (Peña-Sarrionandia et al., 2015). Such theory-informed interpretation is also reflected in personal-resource models that conceptualize EI as relevant to the appraisal and management of work-related strain.

Following this component-level approach, the study examines—within an organizational behavior framework—the associations between emotional intelligence and burnout symptoms using an existing cross-sectional dataset, with particular attention to the differentiated roles of distinct EI components. Drawing on the personal-resource logic of JD–R theory, the study examines whether the observed EI–burnout associations are differentiated at the component level and whether selected indirect statistical patterns are present. The analytical approach, involving hierarchical regression analyses and exploratory bootstrapped indirect-effect models, allows the study to examine theoretically informed associational patterns among EI components and burnout dimensions. These analyses do not establish self-regulatory processes, but they can indicate whether the observed data are consistent with such an interpretation.

The study contributes to the organizational behavior literature in several ways. First, it conceptualizes emotional intelligence not as a general personality trait or trainable competency but as a differentiated personal resource relevant to work-related psychological strain. Second, by applying the component-level structure of the Bar-On model and the multidimensional outcome logic of the BO-SE burnout scale, the study moves beyond dominant global EI–burnout approaches and enables an examination of the functional alignment between emotional resources and distinct burnout symptoms. Third, the research uses exploratory indirect-effect models to examine whether general mood statistically accounts for part of the association between selected EI components and burnout levels. Because the data are cross-sectional, these indirect effects are interpreted as statistical associations rather than as evidence of mediation in a temporal or causal sense. In doing so, the study draws on the personal-resource logic of JD–R theory and contributes to a more precise component-level interpretation of the EI–burnout association. The findings are consistent with a differentiated self-regulatory interpretation, but they do not establish causal mechanisms, temporal ordering, or individual burnout-processing mechanisms.

## 2. Theory and Hypotheses

### 2.1. Burnout as an Individual-Level Indicator of Sustained Organizational Strain

Within the JD–R tradition, burnout is best understood not as a measure of organizational demands but as their individual-level psychological outcome—the depletion of personal resources under sustained work strain (Maslach & Leiter, 2016; Bakker & Demerouti, 2017). This conceptualization is what makes it meaningful to examine the micro-level resources relevant to individual-level responses to prolonged strain even when organizational-level demands are not directly measured (Schaufeli et al., 2020). Accordingly, the JD–R model is used here as an interpretive framework rather than as a model to be tested: because job demands and job resources were not directly measured, the present study does not constitute a test of the full JD–R process.

### 2.2. Emotional Intelligence as an Individual Self-Regulatory Resource

Emotional intelligence (EI) may be viewed as a personal resource for facilitating psychological functioning in the workplace through emotional perception and interpretation.

The findings of the meta-analytic research, which synthesized the literature on emotional intelligence (EI) and emotion regulation, reveal that EI is strongly related to the efficiency of regulating emotional states, thereby providing support for the idea that EI can be conceptualized as a self-regulatory process (Peña-Sarrionandia et al., 2015). From this perspective, EI does not decrease burnout; rather, it is theoretically associated with the appraisal and regulation of emotional responses to work demands, and with the psychological correlates of prolonged strain. In this respect, EI can be conceptualized theoretically as a personal resource related to the internal appraisal and management of work-related strain.

Significant research has established the negative relationship between emotional intelligence (EI) and burnout in different organizational settings. Research conducted among the employee population has found that the level of EI is inversely related to the level of burnout and favorable psychological functioning (Gong et al., 2019). Studies conducted among the teacher population have found that the relationship between EI and burnout occurs not only through direct paths but also through indirect paths, involving social and affective processes (Ju et al., 2015). These findings suggest that it is useful to examine indirect statistical associations among EI components and burnout indicators, while avoiding causal claims about how EI functions in the absence of longitudinal data.

### 2.3. Functional Components of the Bar-On Model and BO-SE Burnout Dimensions

In the present study, emotional intelligence is operationalized based on Bar-On’s model of emotional and social functioning, which distinguishes five broad domains of emotional–social functioning: intrapersonal skills, interpersonal skills, adaptability, stress management, and general mood (Bar-On, 2006). From an organizational behavior perspective, this model can be interpreted as a functionally differentiated system of personal resources that is differentially relevant to the appraisal and management of work-related psychological strain.

Burnout is assessed using the BO-SE questionnaire, which—adapted to educational settings—differentiates four distinct levels of exhaustion: psychological, emotional, physical, and social exhaustion, as well as their aggregated index (Hennig & Keller, 1995). While the BO-SE builds on the Maslach burnout tradition, it provides a more differentiated account of psychological and social forms of depletion in educational contexts (Maslach & Leiter, 2016).

A functional perspective enables the examination of whether specific EI components relate differently to distinct burnout dimensions. Empirical findings support this differentiation: emotional and stress-regulation components tend to show stronger associations with psychological exhaustion, whereas EI elements related to social functioning are more closely linked to social burnout (Ju et al., 2015; Gong et al., 2019).

Based on the personal resource logic of JD–R theory and the functional differentiation of EI, we propose the following hypotheses:

**H1.**  *The overall emotional intelligence score, as conceptualized by the Bar-On model, is negatively associated with all BO-SE burnout dimensions (psychological, emotional, physical, and social) as well as with the aggregated burnout/stress susceptibility index.*

Functionally, the components are expected to align with different facets of strain: intrapersonal skills (self-awareness, self-regard) should be most relevant to the psychological and emotional forms of exhaustion, which involve the monitoring and appraisal of internal states; stress management should act as a transversal regulatory capacity relevant across dimensions; and general mood should be most relevant to the somatic-affective (physical) form. This functional alignment, rather than a uniform negative association, is what H2 predicts.

**H2.**  *The five Bar-On components are differentially associated with the BO-SE burnout dimensions—differing in both the pattern and the strength of their associations—and they differ in their relative explanatory contribution beyond demographic and work-related controls; components most closely tied to self-regulation, particularly stress management, are expected to be the most consistent correlates.*

### 2.4. General Mood as an Exploratory Indirect-Effect Variable

The following two questions are examined as exploratory, supplementary analyses rather than as confirmatory hypotheses. Given the cross-sectional, single-source design, they describe whether the observed associations are consistent with theory-informed indirect-effect patterns; they do not test mediation.

The relationship between emotional intelligence and burnout may involve affective components, but such processes cannot be established with cross-sectional data. Research on emotion regulation indicates that individuals differ in how they perceive, interpret, and regulate emotional states, and these differences are theoretically relevant to work-related strain (Peña-Sarrionandia et al., 2015). Within the Bar-On model, general mood represents a broad affective component related to optimism, positive affect, and perceived emotional well-being (Bar-On, 2006). For this reason, it is theoretically relevant to examine whether general mood statistically accounts for part of the association between other EI components and burnout dimensions.

At the same time, this interpretation requires caution. General mood is conceptually close to well-being and may overlap with the affective content of burnout symptoms. Therefore, in the present study, general mood is treated not as a confirmed psychological mechanism but as an exploratory indirect-effect variable. Empirical studies among teachers suggest that EI may be associated with burnout through indirect statistical patterns rather than only through direct associations, often involving social or affective resources (Ju et al., 2015). In the present study, such models are treated as theory-informed but non-causal. The use of bootstrapped indirect-effect analysis follows the statistical logic of mediation modelling (Hayes, 2018), but the cross-sectional design does not allow conclusions about temporal ordering or causal mediation.

Accordingly, we propose:

**H3.**  *The associations of stress management and adaptability with BO-SE burnout dimensions are expected to be partly statistically accounted for by general mood. These indirect effects are interpreted as exploratory and associational rather than as evidence of causal mediation.*

### 2.5. Intrapersonal Skills, Stress Management, and Emotional Exhaustion (Exploratory)

Within the Bar-On framework, intrapersonal skills refer to emotional self-awareness, self-understanding, and the capacity to recognize one’s internal emotional states, whereas stress management refers to perceived stress tolerance and impulse control (Bar-On, 2006). These components are conceptually related because the recognition of internal emotional states may be associated with perceived capacity to manage stress. However, the temporal ordering of these components cannot be established in the present cross-sectional design.

The personal-resource extension of the JD–R framework suggests that individual resources may be interrelated rather than entirely independent, and that such resources are relevant to the appraisal and management of work-related strain (Bakker & Demerouti, 2017). Previous research has also suggested that emotional intelligence is associated with lower occupational stress and burnout, partly because emotion-related capacities are linked to adaptive responses in demanding situations (Mikolajczak et al., 2007; Szczygieł & Mikolajczak, 2018). These findings provide a theoretical rationale for examining the association among intrapersonal skills, stress management, and emotional exhaustion. They do not, however, imply that the present study can demonstrate a self-regulatory sequence.

Emotional exhaustion is a central dimension of burnout and is closely connected to sustained work-related strain (Maslach & Leiter, 2016). From a theoretical perspective, intrapersonal skills and stress management may both be relevant to this form of exhaustion. The present study therefore examines whether stress management statistically accounts for part of the association between intrapersonal skills and emotional exhaustion. This model is consistent with a differentiated self-regulatory interpretation, but it does not establish causal mechanisms, temporal ordering, or burnout prevention.

Accordingly, we propose:

**H4.**  *The association between intrapersonal skills and emotional exhaustion is expected to be partly statistically accounted for by stress-management capacity. This indirect association is interpreted as theory-informed and exploratory, not as evidence of burnout prevention or causal mediation.*

**Figure 1:**
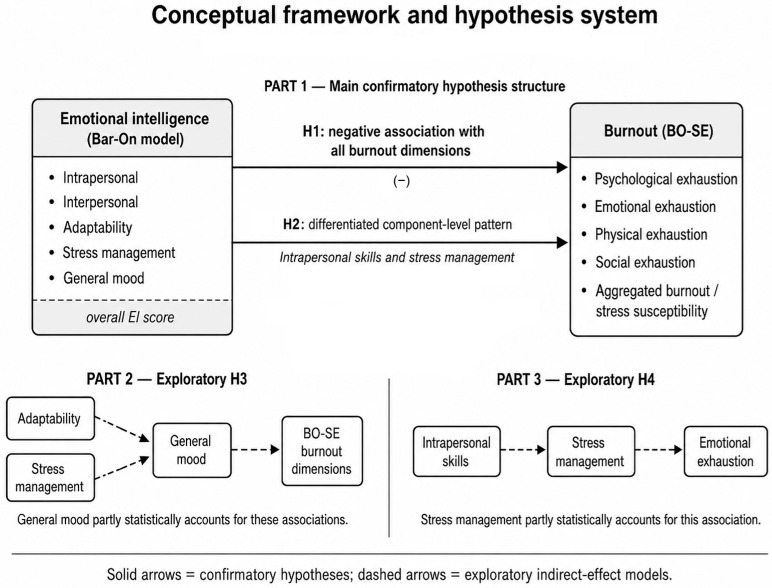
Conceptual framework of the hypothesized component-level associations and exploratory indirect-effect models linking emotional intelligence components and burnout dimensions. Arrows denote hypothesised associations, not causal paths.

Figure 1 illustrates the conceptual framework of the study, presenting the hypothesized component-level associations and exploratory indirect-effect models linking emotional intelligence components to multidimensional burnout outcomes within the personal-resource perspective of the Job Demands–Resources (JD–R) framework.

## 3. Method

### 3.1. Sample and Data Collection

Higher-education instructors are a theoretically apt population for this question: their work combines high and chronic emotional demands with substantial autonomy, a configuration in which personal (rather than purely job-level) resources are expected to be especially consequential for strain (Bakker & Demerouti, 2017). The study employed a cross-sectional survey design conducted among higher-education instructors. A total of 306 participants initially took part in the data collection. Following data screening procedures, cases with incomplete responses were excluded, resulting in a final analytical sample of 292 respondents.

Data were collected in 2025–2026 from three higher-education institutions in Hungary, using convenience and snowball sampling via paper-based questionnaires. The sample comprised 129 men (44.2%) and 163 women (55.8%). The age distribution was heterogeneous: 4% of respondents were younger than 25 years, 26% were between 26 and 35 years, 28% between 36 and 45 years, 24% between 46 and 55 years, and 17% were older than 56 years. The variation in the age and gender of the participants ensured sufficient diversity to examine the relationship between emotional intelligence and the dimensions of burnout. On average, respondents had 18.2 years of experience in higher education (SD = 11.0); they taught 4.6 courses per semester, and the average class size was 27.3 students.

Data collection instruments used were questionnaires administered in educational institutions in paper format. Participation in the study was voluntary and anonymous. Prior information to the participants was given regarding the purpose of the study and how the data collected would be handled. The study protocol complied with institutional ethical standards for anonymous and voluntary survey research. Participants provided informed consent prior to participation.

### 3.2. Measures

#### 3.2.1. Emotional Intelligence

Emotional intelligence was assessed using the Bar-On Emotional-Social Intelligence model (Bar-On, 2006). Emotional intelligence is a multidimensional psychological construct combining capabilities associated with the perception, regulation, and adaptive use of emotions. This model is relevant in organizational settings with persistent emotional demands and stress.

The Bar-On instrument has been widely applied in organizational and educational research. The Bar-On EQ-i comprises 121 items mapping onto five components (intrapersonal, interpersonal, stress management, adaptability, general mood) and 15 subscales; responses were recorded on a Likert-type scale (observed range 0–5). Hungarian empirical studies conducted in educational and organizational samples have consistently confirmed the instrument’s internal reliability and suitability for research purposes. Based on Hungarian validation studies (e.g., Kun, 2011; Kun et al., 2012; Balázs, 2020; Bar-On, 2006), Cronbach’s α values for the overall EI index typically range between 0.88 and 0.94, while reliability estimates for the five main components range between 0.70 and 0.86. At the subscale level, moderate variability has been reported (approximately 0.62–0.82), which is consistent with international validation findings (Dawda & Hart, 2000; Parker et al., 2001; Van Rooy & Viswesvaran, 2004). In the present sample, internal consistency was good to excellent for all five components (Cronbach’s α = 0.92 for intrapersonal, 0.87 for interpersonal, 0.81 for stress management, 0.86 for adaptability, and 0.82 for general mood).

Exploratory and confirmatory factor analyses conducted on Hungarian samples have occasionally indicated partial deviations from the classical Bar-On structure, particularly between adaptability and emotional self-regulation components. These findings support a culturally and contextually sensitive, functional interpretation of the construct rather than indicating diminished validity of the instrument.

In the present study, both the five main EI component scores (intrapersonal skills, interpersonal skills, adaptability, stress management, and general mood) and the overall EI score were included in the analyses, consistent with the model’s self-regulatory and personal resource interpretation and with empirical approaches to burnout processes (Ju et al., 2015; Balázs, 2020).

#### 3.2.2. Burnout

Burnout was assessed with the BO-SE (Burnout–Stress) questionnaire, a teacher-oriented instrument originally developed by Hennig and Keller (1995) within the Maslach-type tradition of measuring exhaustion-based burnout. The questionnaire comprises 24 items that load onto four burnout dimensions—psychological (mental), emotional, physical, and social—with six items each, distributed in an interleaved order across the instrument. Items are rated on a five-point frequency scale (0 = never, 1 = rarely, 2 = sometimes, 3 = often, 4 = always). Each dimension score is the sum of its six items (range 0–24), and the four dimensions together form an aggregate burnout/stress-proneness index (range 0–96). The Hungarian-language version has been used and psychometrically examined in Hungarian teacher samples by Jagodics and Szabó (2014), who reported an acceptable four-factor confirmatory structure (CFI = 0.87, TLI = 0.85, RMSEA = 0.07) and subscale reliabilities ranging from α = 0.62 to 0.82. Reliabilities for the present sample are reported in Section 4.1.

In the present sample, reliability was acceptable for the mental (α = 0.71), emotional (α = 0.76), and physical (α = 0.75) dimensions and high for the aggregated index (α = 0.90); the social dimension showed lower reliability (α = 0.61) and is interpreted with caution. Both Hungarian and international applications have confirmed that the BO-SE is sensitive in differentiating distinct aspects of burnout and is suitable for organizational behaviour research, particularly when burnout is conceptualized as an indicator of individual-level psychological consequences of sustained job demands. Accordingly, in the present study, the BO-SE scales were analysed both as separate dimensions and as an aggregated index in relation to emotional intelligence and personal resources (Maslach & Leiter, 2016; Balázs, 2020).

Although the present study applies the teacher-specific BO-SE instrument, it is important to acknowledge that recent international developments increasingly advocate the use of the Burn-out Assessment Tool (BAT). The BAT conceptualizes burnout as a hierarchical syndrome in which emotional and cognitive impairment constitute core symptoms (Schaufeli et al., 2020). This conceptualization shows theoretical convergence with the multidimensional logic underlying the BO-SE dimensions.

#### 3.2.3. Control Variables

Gender, age, and institutional tenure (years at the current institution) were included as control variables. The originally intended control, length of professional experience, was unusable owing to extensive missingness; institutional tenure, which was essentially complete in the data, was therefore used instead (Schaufeli et al., 2020).

### 3.3. Analytical Strategy

Data analyses were conducted using SPSS Statistics version 25 and jamovi software version 2.7.30, based on the lavaan package (version 0.6-21). Descriptive statistics and Pearson correlations were computed first. Because item-level missingness varied across variables, listwise deletion yields different analytic samples across models; we therefore report the exact *N* for each model rather than a single nominal sample size, using a transparent complete-case approach. Complete cases (*N* = 161 for the full models; *N* = 177 for the EI–burnout focal set) did not differ from excluded cases in gender, age, or tenure, but excluded cases reported higher burnout and lower EI. To verify that the complete-case restriction did not bias the results, we re-estimated all models on the full sample (*N* = 292) using multiple imputation (m = 20) and full-information maximum likelihood; the substantive conclusions were unchanged (see Section 4.5). Per-variable missingness is reported in Table 1 (range 0–22.3%; most focal variables below 12%).

Predictors were standardized within each model. To address multiple testing, *p*-values for the component-level coefficients were adjusted with the Benjamini–Hochberg false discovery rate procedure; a sensitivity power analysis indicated that the smallest analytic samples could reliably detect only moderate effects. An a priori power analysis (linear multiple regression, R^2^ increase; α = 0.05, power = 0.80) indicated that detecting the unique contribution of a single predictor required *N* ≈ 55 for a medium effect (f^2^ = 0.15) and *N* ≈ 395 for a small effect (f^2^ = 0.02), while detecting a small overall-model effect across all eight predictors required *N* ≈ 759. The per-model samples (*N* = 161–276) were therefore adequately powered for medium-sized effects but underpowered for small effects. A two-sided alpha = 0.05 was used. Because all focal variables were self-reported at a single occasion, results are interpreted as associations and statistical indirect effects rather than causal processes.

To test the research hypotheses, linear regression analyses were conducted hierarchically. First, the control variables (gender, age, institutional tenure) entered the model at Step 1, then the total EI score, and finally the five Bar-On components. Individual models were specified for each BO-SE dimension and the combined burnout/stress vulnerability index. Alongside the traditional regression coefficients, the relative impact of the EI dimensions was investigated using relative importance analysis, which provides estimates of the proportionate effect size explained by correlated independent variables in multiple regression (Tonidandel & LeBreton, 2011).

Testing the indirect-effect hypotheses involved bootstrapped analyses (PROCESS macro, Model 4; Hayes, 2018) with 5000 resamples. An indirect effect was considered statistically significant if the 95% confidence interval did not include zero.

#### Robustness and Sensitivity Analyses

Because the study used a cross-sectional, self-report design, causal inference is not possible, and the findings are interpreted as associations rather than causal effects. Potential common method variance was examined with Harman’s single-factor test, which is acknowledged to be a weak diagnostic; component-level and indirect-effect models reduce, but do not eliminate, this concern. Reverse-causal and alternative orderings cannot be ruled out and are addressed in the Limitations. As a sensitivity analysis for the complete-case restriction, the component-level models were additionally re-estimated on the full sample (*N* = 292) using multiple imputation (m = 20; pooled with Rubin’s rules) and full-information maximum likelihood (FIML); results are reported in Section 4.5.

The measurement structure was also examined in the present sample. The four-dimensional structure of the BO-SE was tested with a confirmatory factor analysis using categorical (polychoric) indicators, and the inter-correlations among the five Bar-On components were inspected. Discriminant validity between emotional intelligence and burnout was assessed by comparing one- and two-factor models and via the heterotrait–monotrait ratio of correlations (HTMT). These analyses were conducted in Python 3.12.3 using the semopy package (version 2.3.11).

## 4. Results

### 4.1. Descriptive Statistics and Correlations

Prior to hypothesis testing, the measurement structure was examined in the present sample. A confirmatory four-factor model of the BO-SE showed modest overall fit (CFI = 0.68, TLI = 0.64, RMSEA = 0.11 [90% CI 0.10, 0.11], SRMR = 0.09; ordinal estimation, *N* = 227) and the four dimensions were strongly inter-correlated (r = 0.50–0.73; HTMT up to 0.95). At the latent level, the emotional and physical dimensions were empirically near-indistinguishable (inter-factor correlation ≈ 1.0), so the four-factor structure is retained as the theoretically specified basis for computing the subscale scores rather than as a discriminately validated structural model of burnout. The five Bar-On components were likewise strongly inter-correlated (r = 0.49–0.76), consistent with the well-documented general factor of the instrument and were therefore retained as correlated facets whose unique contributions are examined through the regression and relative-importance analyses reported below. Emotional intelligence and burnout were nonetheless empirically distinguishable: a two-factor model fitted significantly better than a one-factor model (Δχ^2^(1) = 61.0, *p* < 0.001) and the between-domain HTMT was 0.84, indicating that the two constructs, although substantially correlated (latent r = −0.83), are distinct. The two-factor measurement model also showed acceptable residual fit (SRMR = 0.076).

In the first step, descriptive statistics and Pearson correlation coefficients were computed to examine associations between emotional intelligence components and the different burnout dimensions. Correlations among the focal variables are summarized in Table 2.

At the correlational level, all five Bar-On components were examined in relation to the burnout dimensions (Table 2). The multivariate regression and indirect-effect analyses reported below assess their independent and relative contributions. The results indicated that all examined emotional intelligence components were significantly and negatively associated with psychological, emotional, physical, and social burnout, as well as with the aggregated burnout/stress susceptibility index (*p* < 0.01). The magnitude of the correlations ranged from moderate to strong. Particularly strong associations emerged between intrapersonal skills and mental exhaustion (r = −0.66), and between intrapersonal skills and the aggregated burnout index (r ≈ −0.64).

These findings suggest that psychological vulnerability related to burnout is most closely connected to intrapersonal functioning, with broadly similar negative associations across components. At a preliminary level, the correlational results provide support for Hypothesis 1, indicating that emotional intelligence is negatively associated with burnout.

### 4.2. Regression Analyses: Main Effects of Emotional Intelligence (H1)

To test Hypotheses 1 and 2, linear regression analyses were conducted with the distinct burnout dimensions and the aggregated burnout susceptibility index as dependent variables. In the first set of models, the overall emotional intelligence score was entered as the primary predictor.

The results showed that the overall EI score had a significant negative effect on all burnout outcomes (Table 3). The fit of the models varied for the different dimensions of burnout, with adjusted R^2^ ranging from 0.27 to 0.56. In terms of effect size, these findings are considered large in the context of organizational behavior research, suggesting that EI does indeed contribute to a notable extent to the prediction of lingering exhaustion at work.

These findings are consistent with Hypothesis 1, indicating that overall emotional intelligence is negatively associated with all burnout dimensions.

### 4.3. Component-Level Effects and Indirect-Effect Patterns

#### 4.3.1. Component-Level Predictive Patterns (H2)

Hypothesis 2 proposed that the five main components of the Bar-On emotional intelligence model would be differentially associated with the BO-SE burnout dimensions—differing in pattern and strength—following a specific functional alignment (intrapersonal skills for psychological and emotional exhaustion, stress management across dimensions, and general mood for physical exhaustion). This expectation was largely supported.

Table 4 reports the component-level coefficients. The pattern was differentiated: intrapersonal skills were the strongest correlates of mental and emotional exhaustion, stress management was significant across all dimensions except social exhaustion, general mood was specific to physical exhaustion, and adaptability showed no unique effect.

None of the components showed a uniform effect across all outcomes, consistent with the differentiated pattern predicted by Hypothesis 2. The limited effects of interpersonal skills and adaptability align with this differentiation rather than counter it. One expectation was refined: intrapersonal skills proved as consistent a correlate as stress management, and stress management was not significant for social exhaustion.

It is important to emphasize that the standardized regression coefficients presented in Table 4 were derived from simultaneous component-level models in which the five main EI components were entered as parallel predictors within the same regression equation.

These coefficients primarily reflect the relative predictive role of each EI component in relation to the different burnout dimensions with demographic controls (gender, age, tenure) included.

#### 4.3.2. Relative Explanatory Power of Emotional Intelligence Components (H2)

Beyond the absolute coefficients in Table 4, the relative contribution of each component to explained variance was estimated with relative-importance analysis (Tonidandel & LeBreton, 2011); the increment in explained variance contributed by the EI block over the controls (ΔR^2^) was also evaluated (Table 5).

In the first block of the analysis, control variables (age, gender, and tenure within the institution) were entered into the models. In the second block, the five main components of emotional intelligence were introduced simultaneously.

Assessment of multicollinearity confirmed the adequacy of the data for regression analysis: variance inflation factor (VIF) values ranged between 1.06 and 4.20 across models, remaining well below the conservative threshold of 5.

The results indicated that the inclusion of EI dimensions led to a significant increase in explanatory power (ΔR^2^) beyond the control variables for all four burnout outcomes and the aggregate index (ΔR^2^ range: 0.259–0.578; *p* < 0.001). However, the pattern of component effects varied across dimensions. These results are consistent with Hypothesis 2 and refine its specific emphasis, with stress management and intrapersonal skills as the most consistent correlates across dimensions.

#### 4.3.3. Exploratory Indirect Effects Involving General Mood (H3, Supplementary Analysis)

These indirect-effect analyses were not part of the confirmatory analysis plan; they are reported as exploratory, supplementary analyses to characterize the association structure and are not tests of mediation. Hypothesis 3 proposed that the associations of stress management and adaptability with burnout dimensions would be partly statistically accounted for by general mood. The exploratory indirect-effect models yielded dimension-specific patterns (Table 6).

For mental exhaustion, the indirect associations through general mood were statistically significant for both stress management and adaptability.

For emotional and physical exhaustion, the indirect effects through general mood were also statistically significant, although smaller in magnitude.

By contrast, for social exhaustion, the indirect effect through general mood was not statistically significant in the adaptability model. Overall, this pattern is consistent with Hypothesis 3 at the level of exploratory statistical indirect effects.

Overall, the exploratory indirect-effect analyses provide partial and dimension-specific associational support for Hypothesis 3. The indirect effects involving general mood were more evident for mental, emotional, physical, and burnout-proneness outcomes than for social exhaustion, but they should not be interpreted as evidence of causal mediation.

### 4.4. Exploratory Indirect Association Between Intrapersonal Skills and Emotional Exhaustion (H4, Supplementary Analysis)

These indirect-effect analyses were not part of the confirmatory analysis plan; they are reported as exploratory, supplementary analyses to characterize the association structure and are not tests of mediation. To examine Hypothesis 4 as an exploratory indirect-effect model, we applied Hayes’ (2018) PROCESS macro (Model 4) with 5000 bootstrap resamples.

In the model, intrapersonal skills were specified as the independent variable (X), stress management as the exploratory indirect-effect variable (M), and emotional exhaustion as the dependent variable (Y). Gender, age, and tenure were included as control variables (*N* = 211). The results are presented in Table 7.

The model yielded a statistically significant indirect association consistent with the proposed ordering of variables. Intrapersonal skills were positively associated with stress-management capacity (path a: b = 0.54, *p* < 0.001), and stress management, controlling for intrapersonal skills, was negatively associated with emotional exhaustion (path b: b = −0.34, *p* < 0.001).

The bootstrap procedure indicated a significant indirect effect (completely standardized indirect = −0.16; 95% CI [−0.27, −0.11]). Because the direct association between intrapersonal skills and emotional exhaustion remained statistically significant after including stress management, the pattern indicates a statistically significant indirect association alongside a remaining direct association. The full model explained 48% of the variance in emotional exhaustion (R^2^ = 0.480, *p* < 0.001).

These findings are consistent with Hypothesis 4 in an associational sense: higher intrapersonal skills were related to lower emotional exhaustion, and part of this relationship was statistically accounted for by stress management.

### 4.5. Robustness and Sensitivity

Because the design is cross-sectional and based on self-report, common method variance cannot be excluded; Harman’s single-factor test, a weak diagnostic, indicated a large general factor (see Methods and Limitations). The bootstrapped indirect effects reported in Section 4.4 were consistent in sign and approximate magnitude with the regression results and are interpreted as associational.

To assess whether listwise deletion biased the results, we re-estimated the component-level models on the full sample (*N* = 292) using multiple imputation (m = 20, pooled with Rubin’s rules) and full-information maximum likelihood (FIML). Across both approaches, the substantive pattern was reproduced (Table 8): stress management remained a significant negative correlate of mental, emotional, and physical exhaustion, intrapersonal skills remained specifically associated with mental and emotional exhaustion, and adaptability showed no unique effect. Two peripheral coefficients were sample-sensitive (stress management → social exhaustion and general mood → physical exhaustion), consistent with the lower reliability of the social dimension. Notably, the small positive intrapersonal–physical coefficient observed under listwise deletion did not persist under FIML or multiple imputation, indicating that it reflected the reduced complete-case sample rather than a substantive effect. Overall, the main conclusions did not depend on the complete-case restriction.

### 4.6. Summary of Results

Overall, the results are consistent with emotional intelligence being negatively associated with burnout in a differentiated rather than homogeneous way. The component-level analyses indicate that intrapersonal skills and stress management were the two most consistent correlates across burnout dimensions, with complementary coverage: stress management was most consistent for mental, emotional, and physical exhaustion, whereas intrapersonal skills showed the strongest associations with mental and emotional exhaustion. Adaptability did not contribute uniquely once the other components were considered.

**Figure 2:**
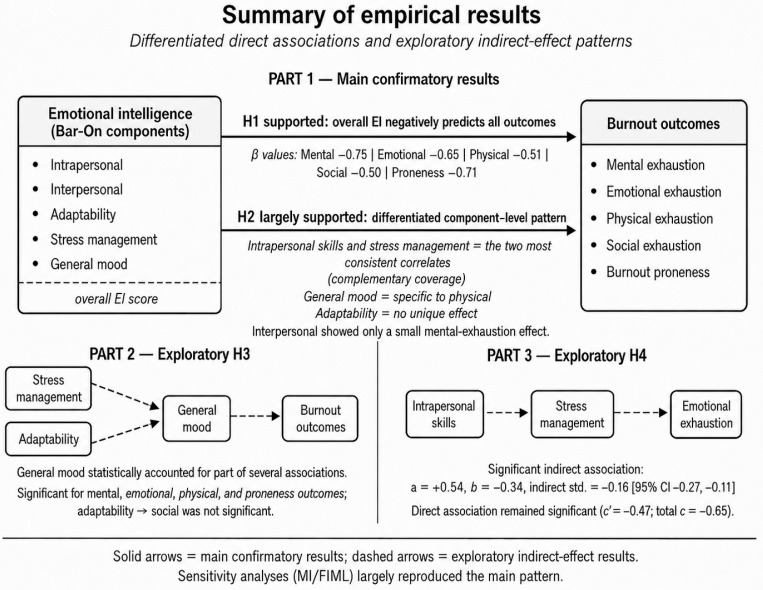
Summary of the main component-level associations and exploratory indirect-effect patterns linking emotional intelligence components and burnout dimensions. Arrows denote associations, not causal effects.

Figure 2 summarizes the differentiated direct associations and exploratory indirect-effect patterns integrating the main regression and indirect-effect results.

As illustrated in Figure 2, the findings are consistent with a differentiated component-level interpretation of emotional intelligence in relation to burnout dimensions. The most consistent negative associations were observed for stress management and intrapersonal skills, with intrapersonal skills showing particular relevance for the mental and emotional forms of exhaustion.

## 5. Discussion

This study examined how emotional intelligence is associated with different burnout dimensions among higher-education instructors, with specific attention to the differentiated roles of EI components. The findings contribute to the literature by showing that a component-level interpretation of EI may provide a more differentiated account of burnout-related variation than a single global EI score. This contribution is theoretical and associational rather than causal. In interpreting the results, it is essential to emphasize that the study used a cross-sectional, single-source self-report design. The findings are consistent with a differentiated self-regulatory interpretation, but they do not establish causal mechanisms, temporal ordering, or the development of burnout over time. The results are consistent with the personal-resource perspective within the JD–R model, in the limited sense that EI components were negatively associated with burnout indicators. Because job demands and job resources were not directly measured, the present study should not be interpreted as a test of the full JD–R process (Bakker & Demerouti, 2017; Lesener et al., 2019). Recent studies on teachers provide useful context for these results, including work on resilience and emotionally demanding teaching settings (Lu et al., 2024; Geraci et al., 2023). However, the present study does not test these broader processes directly. More narrowly, the findings suggest that EI may be more informative when examined at the component level than only as a global score, and that different EI components are differentially associated with burnout dimensions among higher-education instructors.

One of the main theoretical implications of this study is that emotional intelligence may be more informative when examined at the component level rather than only as a global score. The results indicate that EI components were not uniformly associated with all burnout indicators. Intrapersonal skills and stress management were the two most consistent correlates: intrapersonal skills were most strongly associated with mental and emotional exhaustion (but were also relevant to social exhaustion in the full sample), stress management was consistent across the mental, emotional, and physical dimensions, and general mood was specifically associated with physical exhaustion. This differentiated pattern should be interpreted as variation in the relative strength of associations among correlated constructs, not as evidence of distinct burnout phases or a confirmed self-regulatory sequence.

These findings are broadly consistent with emotion-regulation perspectives, which emphasize that individuals differ in how they perceive, interpret, and manage emotional experiences (Gross, 2015; Cludius et al., 2020). They may also be interpreted in light of resource-depletion perspectives on sustained work strain (Hockey, 2013; Baumeister et al., 2018). However, the present study did not measure specific emotion-regulation strategies such as cognitive reappraisal, nor did it assess resilience, psychological capital, or resource depletion directly. Therefore, these constructs are best understood as theoretical contexts for interpreting the observed associations rather than as mechanisms demonstrated by the data. Similarly, although previous research has linked cognitive reappraisal and emotion regulation to teacher well-being and teaching effectiveness (Riepenhausen et al., 2022; Aldrup et al., 2024), the present findings do not test these processes directly. This interpretation is also compatible with recent studies showing that emotion regulation, resilience, self-efficacy, and socio-emotional competence are associated with burnout-related outcomes in educational settings (Li, 2023; Ornaghi et al., 2023). These studies provide useful context for the present findings, but they should not be taken as evidence that the same processes were directly tested here. The current results contribute more narrowly by showing that EI components may provide a differentiated account of burnout-related variation when examined as correlated personal-resource indicators rather than as a single aggregated construct.

Because the design is cross-sectional, reverse and reciprocal relationships cannot be excluded and are considered in the Limitations.

## 6. Theoretical Implications

The following interpretations are theoretical: constructs such as cognitive reappraisal, resilience, and resource depletion were not directly measured here and are invoked only to interpret the observed associations. The findings of the present study contribute to the emerging literature in organizational behavior and work psychology at several important levels, particularly in advancing a component-level understanding of personal resources.

### 6.1. Refining the Personal-Resource Perspective Within the JD–R Framework

The findings refine the personal-resource perspective within the JD–R framework by suggesting that EI components are not uniformly associated with burnout dimensions. Stress management and intrapersonal skills showed the most consistent negative associations, with complementary coverage across dimensions, whereas general mood was more dimension-specific. This pattern does not demonstrate hierarchical resource interactions, but it suggests that component-level EI analysis may provide greater theoretical precision than global EI scores.

The association between intrapersonal skills, stress management, and emotional exhaustion is consistent with a self-regulatory interpretation, but the present design does not establish that intrapersonal awareness precedes stress management or that stress management reduces later burnout. Theoretical claims about antecedent and executive resources should therefore be understood as hypotheses for future longitudinal research.

### 6.2. Functional Differentiation in EI–Burnout Associations

Second, the component-level analyses highlight the functional differentiation of the EI–burnout associations. The results suggest that the five Bar-On components are not interchangeable in their relationships with burnout indicators. Intrapersonal skills and stress management showed the most consistent negative associations, with complementary coverage: intrapersonal skills were most strongly related to mental and emotional exhaustion (and, on the full sample, to social exhaustion), stress management to mental, emotional, and physical exhaustion, and general mood most clearly to physical exhaustion.

This pattern is compatible with process-based emotion-regulation theories (Gross, 2015), but the present study did not measure specific regulation strategies or dynamic regulatory processes. Therefore, the results should be interpreted as component-level associational evidence. They suggest that the EI–burnout relationship is differentiated and context-sensitive, but they do not establish functional emotion-regulation processes.

### 6.3. Burnout Dimensions and Differentiated EI Associations

The differentiated pattern is theoretically informative because the burnout indicators did not relate to EI components in a uniform way. Mental and emotional exhaustion were more strongly associated with intrapersonal skills and stress management, whereas physical exhaustion was also associated with general mood. Social exhaustion showed a more limited pattern; stress management was the most relevant component in the complete-case models, whereas intrapersonal skills emerged as the most relevant component in the full sample (Table 8). Because the social-exhaustion subscale showed lower internal consistency (α = 0.61), its component-level associations are interpreted with particular caution.

These differences should not be interpreted as separate burnout phases, a depletion sequence, or a dynamic self-regulatory process. Rather, they indicate that different BO-SE indicators may show different degrees of association with specific EI components. This interpretation is consistent with the broader view that burnout is multidimensional, but the present study cannot determine whether the observed patterns reflect temporal development, resource loss, or recovery processes.

The findings may also be read in light of Conservation of Resources theory (Hobfoll, 1989; Hobfoll et al., 2018), insofar as EI components can be understood as personal-resource indicators. However, the study did not directly measure resource gain, resource loss, or emotional capital. Accordingly, COR theory is used here as an interpretive framework rather than as an empirically tested process model.

## 7. Practical Implications

The practical implications of the present findings should be regarded as tentative. The study did not test interventions, coaching programs, selection procedures, or organizational well-being strategies. Therefore, the results should not be used *as* direct evidence for burnout prevention or intervention effectiveness.

At most, the findings suggest that institutions concerned with instructor well-being may benefit from distinguishing among burnout dimensions rather than treating burnout as a single undifferentiated outcome. The results also suggest that different EI components may be differentially associated with these dimensions. In practice, this means that assessment and support initiatives should be designed cautiously and evaluated empirically before any firm recommendations are made.

Future intervention studies could examine whether strengthening stress-management or intrapersonal competencies leads to later reductions in burnout symptoms. Such claims, however, remain beyond the scope of the present cross-sectional study.

## 8. Limitations and Future Research Directions

The findings of the present study should be interpreted in light of several methodological and theoretical limitations, which at the same time delineate important avenues for future research. A central interpretive limitation concerns the indirect-effect models. Although the PROCESS analyses produced statistically significant indirect effects in several models, these results do not establish psychological mechanisms, temporal sequences, or causal mediation. The terms “indirect effect” and “mediation” are used here in a statistical sense only. It remains possible that burnout symptoms influence perceived EI, that both are shaped by broader affective disposition, or that shared self-report variance inflates the observed associations.

### 8.1. Methodological Limitations and the Question of Causal Inference

The main limitation of the current study is its cross-sectional nature, which does not allow causal interpretation of the observed relationships. This issue is particularly relevant for Hypothesis 4, where stress management was examined as an exploratory indirect-effect variable in the association between intrapersonal skills and emotional exhaustion. Although the proposed ordering of variables was theoretically informed, it cannot be empirically validated with cross-sectional data. Therefore, the indirect-effect results should be interpreted as statistical patterns rather than as evidence of temporal mediation or a demonstrated self-regulatory process.

It is also possible that the direction of the association is reversed or reciprocal. For example, higher current burnout symptoms may influence how individuals perceive and report their emotional intelligence. This possibility is compatible with the resource-loss logic of Conservation of Resources theory (Hobfoll, 1989), but the present data cannot test such reciprocal or longitudinal processes.

A further limitation concerns cross-sectional indirect-effect analysis more generally: bootstrapped indirect effects can identify whether an indirect statistical pattern is present, but they cannot establish temporal order. Future research should test the proposed ordering among intrapersonal skills, stress management, and emotional exhaustion using larger, pre-registered longitudinal samples and full-information maximum likelihood estimation to reduce the impact of missing data on power. Cross-lagged panel models would be especially useful for examining whether the ordering assumed in the present theory-informed model is supported over time (Selig & Preacher, 2009).

A further limitation concerns the measurement structure itself. Although emotional intelligence and burnout were empirically distinguishable, the five EI components and the four burnout dimensions were strongly inter-correlated and did not form sharply separable factors in the present sample. The differentiated pattern of associations reported here should therefore be read as differences in the relative emphasis of correlated facets rather than as evidence of fully independent constructs or mechanisms. More specifically, the four-factor BO-SE model showed only modest fit in this sample (CFI = 0.68), and the emotional and physical dimensions were empirically near-indistinguishable; however, the component-level findings that constitute the study’s contribution do not depend on this dimensional model, and the aggregate burnout index showed excellent reliability (α = 0.90).

A further caveat concerns the general-mood indirect-effect variable: general mood overlaps conceptually with the affective content of emotional exhaustion, so the observed indirect effects may partly reflect shared affective variance rather than a distinct regulatory process. These indirect-effect findings should therefore be read as associational and provisional.

### 8.2. Common Method Variance and Subjectivity of the Data

The sole use of a data collection method through self-report questionnaires is subject to common method variance (CMV). This is a recognized problem in using this methodology, as discussed by Podsakoff et al. (2003). Although emotional intelligence and burnout are internal psychological constructs, which are more commonly assessed through a self-report methodology, the responses obtained might have been subject to the influences of social desirability effects and/or the affective states of the participants at the time of responding. Future studies should combine self-report measures with additional data sources, such as peer ratings, behavioral indicators, longitudinal diary data, or organizational-level information. Such designs would provide a stronger empirical basis for evaluating whether EI components are related to subsequent changes in burnout symptoms and whether the proposed self-regulatory interpretation is supported over time.

### 8.3. Sampling Characteristics and Generalizability

The composition of the sample population, which consisted specifically of higher-education instructors, limits the generalization of the results to other organizational environments. The teaching profession is known for having exceptionally high levels of emotional labor, and intrapersonal awareness and stress management skills may play particularly significant roles. Thus, the negative associations of specific components of EI may have been overrepresented.

Comparative studies on other professions involving lower levels of emotional labor (e.g., technical and administrative work) should be conducted to assess the relative contribution of the negative associations of EI components to the presence of emotional labor.

### 8.4. Additional Variables and Boundary Conditions for Future Research

Although the present study examined stress management and general mood as exploratory indirect-effect variables, the EI–burnout association is likely to be shaped by additional individual and organizational factors. Future research could incorporate organizational-level resources, such as supervisor support, psychological safety, workload, autonomy, and institutional climate, as potential boundary conditions. Such variables would make it possible to examine whether the associations between EI components and burnout indicators vary across different work environments.

Future studies could also include additional theory-informed variables, such as work–life balance, cognitive appraisal, perceived emotional labor, coping strategies, or recovery experiences. These variables may help clarify when and for whom EI components are associated with lower burnout symptoms. However, such models should be tested with longitudinal or multi-method designs before being interpreted as evidence of causal processes.

Finally, future research should distinguish clearly between statistical indirect effects and putative psychological processes. The present findings identify differentiated associational patterns, but they do not determine whether EI components influence burnout trajectories. Establishing such claims would require repeated measurement, temporal separation of predictors and outcomes, and preferably intervention or quasi-experimental designs.

## 9. Conclusions

Among higher-education instructors, emotional intelligence was negatively associated with burnout, but not uniformly: stress-regulation capacity (stress tolerance and impulse control) and intrapersonal skills were the two broadest correlates across dimensions, with complementary coverage, whereas general mood showed dimension-specific associations, and exploratory indirect-effect models suggested that general mood and stress management statistically accounted for part of these associations. The results are consistent with viewing emotional intelligence as a differentiated set of personal resources rather than as a single global trait. Because the design is cross-sectional and based on self-report, these conclusions remain associational. Future longitudinal, multi-method, and intervention studies are needed to determine whether the observed component-level patterns reflect actual self-regulatory processes or causal effects.

## Figures and Tables

**Table 1:** Missing data by variable (*N* = 292).

Variable	Valid n	Missing n	Missing %
EI–Intrapersonal	258	34	11.60
EI–Interpersonal	264	28	9.60
EI–Stress management	262	30	10.30
EI–Adaptability	266	26	8.90
EI–General mood	274	18	6.20
Burnout–Mental	233	59	20.20
Burnout–Emotional	264	28	9.60
Burnout–Physical	276	16	5.50
Burnout–Social	245	47	16.10
Burnout proneness (sum)	227	65	22.30
Gender	292	0	0.00
Age	269	23	7.90
Institutional tenure	273	19	6.50

**Table 2:** Descriptive statistics and correlations between EI components and burnout dimensions.

Variable	M	SD	Mental	Emotional	Physical	Social	Proneness
Intrapersonal	26.57	3.33	−0.66 **	−0.62 **	−0.37 **	−0.49 **	−0.64 **
Interpersonal	32.01	3.47	−0.60 **	−0.38 **	−0.26 **	−0.37 **	−0.47 **
Stress management	31.84	4.26	−0.48 **	−0.52 **	−0.43 **	−0.41 **	−0.54 **
Adaptability	32.10	3.71	−0.60 **	−0.52 **	−0.40 **	−0.49 **	−0.60 **
General mood	31.81	3.85	−0.62 **	−0.50 **	−0.40 **	−0.44 **	−0.57 **
Mental exhaustion	4.18	3.13	—				
Emotional exhaustion	6.38	3.69	0.67 **	—			
Physical exhaustion	5.84	3.66	0.50 **	0.73 **	—		
Social exhaustion	3.72	3.25	0.58 **	0.67 **	0.56 **	—	
Burnout proneness	20.13	11.68	0.81 **	0.92 **	0.84 **	0.81 **	—

Note. *p* < 0.01; M and SD are shown for each variable; the lower block reports correlations among the burnout dimensions. *N* = 227–292 (pairwise). ** *p* < 0.01.

**Table 3:** Regression of burnout outcomes on overall emotional intelligence (controlling for gender, age, and tenure).

Outcome	*N*	β (Overall EI)	R^2^	Adj. R^2^
Mental	161	−0.75 **	0.57	0.56
Emotional	179	−0.65 **	0.47	0.45
Physical	185	−0.51 **	0.30	0.29
Social	169	−0.50 **	0.28	0.27
Burnout proneness	161	−0.71 **	0.53	0.52

Note. Overall emotional intelligence is a composite index based on the five main Bar-On components (intrapersonal skills, interpersonal skills, stress management, adaptability, and general mood). Values are standardized regression coefficients (β) from multiple linear regression models estimating the total explanatory power of EI for each burnout dimension. Age, gender, and organizational tenure were included as controls in all models but omitted from the table for parsimony. Models use the same listwise-complete sample as Table 4 (*N* = 161–185). ** *p* < 0.01.

**Table 4:** Component-level prediction of burnout dimensions (standardized coefficients with standard errors, confidence intervals, and collinearity diagnostics).

Predictor	β	SE	95% CI	t	*p*	VIF
**Mental—*N* = 161, adj. R^2^ = 0.571, ΔR^2^ (EI block) = 0.578**						
Intrapersonal	−0.454 **	0.079	[−0.61, −0.30]	−5.78	<0.001	2.30
Interpersonal	−0.147 *	0.072	[−0.29, −0.00]	−2.03	0.044	1.96
Stress management	−0.318 **	0.076	[−0.47, −0.17]	−4.19	<0.001	2.15
Adaptability	+0.062	0.096	[−0.13, +0.25]	+0.64	0.522	3.46
General mood	−0.111	0.084	[−0.28, +0.05]	−1.33	0.187	2.61
Age	−0.039	0.061	[−0.16, +0.08]	−0.65	0.519	1.39
Gender (female)	+0.050	0.058	[−0.06, +0.16]	+0.88	0.382	1.24
Tenure	+0.030	0.060	[−0.09, +0.15]	+0.49	0.623	1.36
**Emotional—*N* = 179, adj. R^2^ = 0.475, ΔR^2^ (EI block) = 0.437**						
Intrapersonal	−0.224 **	0.082	[−0.39, −0.06]	−2.72	0.007	2.30
Interpersonal	−0.108	0.076	[−0.26, +0.04]	−1.41	0.160	1.97
Stress management	−0.378 **	0.079	[−0.53, −0.22]	−4.78	<0.001	2.12
Adaptability	−0.017	0.100	[−0.21, +0.18]	−0.17	0.868	3.39
General mood	−0.115	0.088	[−0.29, +0.06]	−1.31	0.192	2.61
Age	−0.046	0.064	[−0.17, +0.08]	−0.72	0.470	1.38
Gender (female)	+0.056	0.060	[−0.06, +0.17]	+0.94	0.348	1.22
Tenure	+0.157 *	0.064	[+0.03, +0.28]	+2.45	0.015	1.39
**Physical—*N* = 185, adj. R^2^ = 0.391, ΔR^2^ (EI block) = 0.366**						
Intrapersonal	+0.192 *	0.088	[+0.02, +0.36]	+2.19	0.030	2.32
Interpersonal	−0.093	0.081	[−0.25, +0.07]	−1.14	0.254	1.99
Stress management	−0.339 **	0.083	[−0.50, −0.18]	−4.09	<0.001	2.08
Adaptability	−0.147	0.105	[−0.35, +0.06]	−1.40	0.162	3.33
General mood	−0.287 **	0.093	[−0.47, −0.10]	−3.10	0.002	2.59
Age	+0.050	0.068	[−0.08, +0.18]	+0.74	0.462	1.38
Gender (female)	+0.148 *	0.064	[+0.02, +0.27]	+2.32	0.021	1.23
Tenure	+0.046	0.068	[−0.09, +0.18]	+0.68	0.496	1.39
**Social—*N* = 169, adj. R^2^ = 0.270, ΔR^2^ (EI block) = 0.259**						
Intrapersonal	−0.119	0.100	[−0.32, +0.08]	−1.19	0.237	2.32
Interpersonal	−0.027	0.094	[−0.21, +0.16]	−0.29	0.773	2.01
Stress management	−0.224 *	0.097	[−0.42, −0.03]	−2.30	0.023	2.18
Adaptability	−0.183	0.123	[−0.43, +0.06]	−1.49	0.138	3.47
General mood	−0.068	0.108	[−0.28, +0.14]	−0.63	0.531	2.66
Age	+0.064	0.078	[−0.09, +0.22]	+0.82	0.412	1.39
Gender (female)	+0.037	0.074	[−0.11, +0.18]	+0.51	0.614	1.26
Tenure	+0.093	0.077	[−0.06, +0.24]	+1.22	0.225	1.35
**Burnout proneness—*N* = 161, adj. R^2^ = 0.552, ΔR^2^ (EI block) = 0.538**						
Intrapersonal	−0.159 *	0.080	[−0.32, −0.00]	−1.98	0.050	2.30
Interpersonal	−0.126	0.074	[−0.27, +0.02]	−1.70	0.092	1.96
Stress management	−0.395 **	0.078	[−0.55, −0.24]	−5.10	<0.001	2.15
Adaptability	−0.095	0.098	[−0.29, +0.10]	−0.96	0.338	3.46
General mood	−0.153	0.085	[−0.32, +0.02]	−1.79	0.075	2.61
Age	+0.016	0.062	[−0.11, +0.14]	+0.26	0.794	1.39
Gender (female)	+0.081	0.059	[−0.04, +0.20]	+1.38	0.169	1.24
Tenure	+0.069	0.062	[−0.05, +0.19]	+1.11	0.268	1.36

Note. β = standardized regression coefficient from OLS models in which the five Bar-On EI components and the three controls were entered simultaneously; predictors and outcomes were standardized (z-scores). 95% CI = 95% confidence interval for β. VIF = variance inflation factor. EI components were computed from the Bar-On subscales (stress management = stress tolerance + impulse control; adaptability = reality testing + flexibility + problem solving). Analyses used listwise-complete cases per model. * *p* < 0.05; ** *p* < 0.01.

**Table 5:** Relative importance (% of model R^2^) of EI components by burnout dimension.

Outcome	Intrapers.	Interpers.	Stress Mgmt	Adaptab.	Gen. Mood	ΔR^2^ (EI Block)
Mental	32.6	15.3	19.6	15.0	17.5	0.58
Emotional	19.1	13.4	32.9	17.9	16.7	0.44
Physical	5.1	12.8	38.2	20.3	23.7	0.37
Social	17.2	10.2	30.3	27.1	15.3	0.26
Burnout proneness	14.9	14.0	33.2	20.4	17.5	0.54

Note: relative importance weights (Tonidandel & LeBreton, 2011) rescaled to 100% of the model R^2^ (controls partialled out). Computed on the same listwise-complete sample as Table 3 and Table 4 (*N* = 161–185).

**Table 6:** Direct associations and exploratory indirect effects of emotional intelligence components on burnout dimensions involving general mood.

Predictor → Outcome	*N*	Direct β	Indirect β	95% CI	Sig.
Stress mgmt → Mental	195	−0.36	−0.21	[−0.29, −0.13]	yes
Stress mgmt → Emotional	223	−0.39	−0.14	[−0.23, −0.06]	yes
Stress mgmt → Physical	233	−0.33	−0.12	[−0.19, −0.05]	yes
Stress mgmt → Social	207	−0.34	−0.12	[−0.21, −0.05]	yes
Stress mgmt → Proneness	195	−0.45	−0.15	[−0.23, −0.08]	yes
Adaptability → Mental	195	−0.44	−0.19	[−0.30, −0.10]	yes
Adaptability → Emotional	223	−0.40	−0.14	[−0.24, −0.05]	yes
Adaptability → Physical	233	−0.29	−0.14	[−0.24, −0.05]	yes
Adaptability → Social	207	−0.47	−0.06	[−0.17, 0.04]	ns
Adaptability → Proneness	195	−0.51	−0.12	[−0.22, −0.04]	yes

Note. Values represent standardized regression coefficients (β). Indirect effects are completely standardized statistical indirect effects estimated using PROCESS Model 4 with 5000 bootstrap samples. An indirect effect was treated as statistically significant when the 95% bootstrap confidence interval did not include zero. All models controlled for age, gender, and organizational tenure.

**Table 7:** Exploratory indirect-effect model involving intrapersonal skills, stress management, and emotional exhaustion (*N* = 211).

Path	b	SE	*p*
Intrapersonal → Stress management (a)	0.54	0.08	<0.001
Stress management → Emotional exhaustion, controlling X (b)	−0.34	0.05	<0.001
Intrapersonal → Emotional exhaustion, direct (c′)	−0.47	0.06	<0.001
Intrapersonal → Emotional exhaustion, total (c)	−0.65	0.06	<0.001
Indirect (a × b), std. = −0.16	—	—	95% CI [−0.27, −0.11]

Note. The table presents unstandardized regression coefficients (b), following the conventions of PROCESS Model 4. Bootstrap confidence intervals for the statistical indirect effect were generated based on 5000 resamples. The model included gender, age, and tenure as control variables. The completely standardized indirect effect was −0.16 (95% CI [−0.27, −0.11]).

**Table 8:** Sensitivity of component-level standardized coefficients to missing-data handling: complete-case (listwise) estimates versus multiple imputation and FIML on the full sample.

Predictor	Listwise β	MI β (*N* = 292)	FIML β (*N* = 292)
**Mental exhaustion**			
Intrapersonal	−0.454 **	−0.360 **	−0.337 **
Interpersonal	−0.147 *	−0.203 **	−0.190 **
Stress management	−0.318 **	−0.187 **	−0.179 **
Adaptability	+0.062	−0.023	−0.043
General mood	−0.111	−0.086	−0.098
**Emotional exhaustion**			
Intrapersonal	−0.224 **	−0.366 **	−0.350 **
Interpersonal	−0.108	−0.061	−0.044
Stress management	−0.378 **	−0.276 **	−0.285 **
Adaptability	−0.017	+0.009	−0.012
General mood	−0.115	−0.046	−0.054
**Physical exhaustion**			
Intrapersonal	+0.192 *	−0.045	−0.028
Interpersonal	−0.093	+0.050	+0.056
Stress management	−0.339 **	−0.257 **	−0.227 **
Adaptability	−0.147	−0.077	−0.100
General mood	−0.287 **	−0.203 *	−0.230 **
**Social exhaustion**			
Intrapersonal	−0.119	−0.208 *	−0.208 **
Interpersonal	−0.027	−0.080	−0.082
Stress management	−0.224 *	−0.129	−0.136
Adaptability	−0.183	−0.163	−0.167
General mood	−0.068	−0.036	−0.022
**Burnout proneness (aggregate)**			
Intrapersonal	−0.159 *	−0.307 **	−0.288 **
Interpersonal	−0.126	−0.089	−0.065
Stress management	−0.395 **	−0.275 **	−0.268 **
Adaptability	−0.095	−0.066	−0.078
General mood	−0.153	−0.067	−0.101

Note. β = standardized regression coefficient; each model includes the five Bar-On EI components and gender, age, and tenure. Listwise = complete-case estimates (identical to Table 4; *N* = 161–185). MI = multiple imputation (m = 20, pooled via Rubin’s rules; *N* = 292). FIML = full-information maximum likelihood (*N* = 292). * *p* < 0.05; ** *p* < 0.01.

## Data Availability

The data presented in this study are available from the corresponding author upon reasonable request. The data are not publicly available due to privacy and ethical restrictions.
